# The Influence of Selected Plant Essential Oils on Morphological and Physiological Characteristics in *Pseudomonas Orientalis*

**DOI:** 10.3390/foods8070277

**Published:** 2019-07-23

**Authors:** Katarzyna Leja, Kamila Szudera-Kończal, Ewa Świtała, Wojciech Juzwa, Przemysław Łukasz Kowalczewski, Katarzyna Czaczyk

**Affiliations:** 1 Department of Biotechnology and Food Microbiology, Poznań University of Life Sciences, 48 Wojska Polskiego St, 60-627 Poznań, Poland; 2 Institute of Food Technology of Plant Origin, Poznań University of Life Sciences, 31 Wojska Polskiego St, 60-624 Poznań, Poland

**Keywords:** antibacterial activity, antibacterial mode of action, plant essential oils, *Pseudomonas orientalis* isolates

## Abstract

The aim of this work was to estimate the antibacterial activity of selected essential oils on *Pseudomonas orientalis* strains isolated from foods. An attempt was also made to identify the mechanisms of the action of the plant oils. Classical methods of assessment of the effectiveness of antimicrobial activity of oils were linked with flow cytometry. It was observed that bergamot, lemongrass, bitter orange, juniper, and black pepper oils have bacteriostatic effect against *P. orientalis* P49. *P. orientalis* P110 is sensitive to lime, lemongrass, juniper, rosemary, and black pepper oils. Additionally, plant oils with biostatic effect on *P. orientalis* limited the intracellular metabolic activity of cells; this was closely linked with the ability of plant oils’ bioactive components to interact with bacteria cell membrane, causing the release of membrane proteins. As a result, the selective permeability of the cell membranes were damaged and the bacterial shape was transformed to coccoid in form.

## 1. Introduction

Although the rapid development of chemical and medical sciences in the 20th century significantly weakened the importance of essential oils, there has recently been renewed interest in the huge antimicrobial potential of natural plant oils [1,2,3]. Contemporary food market trends, associated with consumer expectations, are directed at natural products of high quality, low processing and long shelf-life [4,5]. The current fashion for healthy nutrition and care for the natural environment make it necessary to replace chemical preservatives with naturally-formed ones [6,7,8].

Essential oils are produced by plants as secondary metabolites. They are mixtures of aromatic compounds, including terpenes, terpenoids, phenols, and rakebones [9,10]. Essential oils, due to their huge biological activity, are used, among others, in the food industry as natural preservatives in order to reduce or eliminate the addition of preservative chemicals to foods such as cereals, grains, pulses, fruits, and vegetables [11,12].

The main advantage of using essential oils in very low concentrations as preservatives is that, unlike for chemical preservatives, even their long-term use does not cause the activation of resistance mechanisms in bacterial cells. Essential oils decrease the metabolic activity of cells through their influence on the phospholipid bilayer of the cell membrane (interaction with proteins in cell), decreasing ATP synthesis, changing the pH gradient across the cytoplasmic membrane, inhibiting *quorum sensing* action, as well as destruction or inactivation of genetic material. However, they do not cause cell death. Thus, such plant-derived substances are considered to be safe alternatives to synthetic ones [13,14]. Moreover, essential oils frequently demonstrate bacteriostatic effects comparable to or even stronger than such synthetic preservatives [15,16,17,18]. 

*Pseudomonas orientalis* are Gram-negative, aerobic bacteria. This species has been isolated for the first time from spring water in Lebanon. *P. orientalis* are able to grow in protein-rich food products (like fish) but they do not convert to carbohydrates. Moreover, they have the ability to multiply in water which is poor in organic carbon compounds, and hence, *P. orientalis* contamination can pose a significant risk to the food industry [19]. In the literature, there is very little information regarding the metabolic abilities of these bacteria. As a result, the authors of this study began their investigations by analyzing the physical and biochemical properties of the studied *P. orientalis* strains and, simultaneously, the inhibitory influence of essential oils in concentration on these properties [16].

The available literature provides only limited data about the complete mode of action of plant essential oils against *Pseudomonas* associated with foods [20]. Furthermore, the literature also lacks data on the antibacterial effect of oils on *P. orientalis* bacteria. Therefore, the aim of this work was to comprehensively evaluate the antimicrobial activity of selected essential oils on two *P*. *orientalis* strains, designated as P49 and P110, isolated from two different raw salmon fillets in the Department of Biotechnology and Food Microbiology, Poznań University of Life Sciences. The authors deliberately chose these bacteria for testing because they are typical saprophytic strains living in fish. Especially in light of increasing raw fish consumption, such as in sushi, the inhibition of such bacterial growth is a very important issue.

## 2. Materials and Methods

### 2.1. Bacteria Strains

*P. orientalis* P49 and P110 were isolated from raw salmon fillets. Stocks of *P. orientalis* are kept at culture collection of Department of Biotechnology and Food Microbiology at Poznań University of Life Sciences. The strains were identified using the 16S rRNA technique.

### 2.2. Essential Oils

Bergamot (*Citrus bergamium*), nutmeg (*Myristica fragrans*), bitter orange (*Citrus aurantium* L.), lime (*Citrus aurantifolia*), lemongrass (*Cymbopogon citratus*), juniper (*Juniperus communis* L.), black pepper (*Piper nigrum*), and rosemary oils (*Rosmarinus officinalis*) were obtained from P.P.H Vera-Nord Company (Stanisławów Drugi, Poland). St. John’s wort (*Hypericum perforatum*) oil was purchased from Bamer Company (Włocławek, Poland). Analytic standards originated from Sigma-Aldrich (Poznań, Poland). The essential oils were stored at 4 °C in darkness before use, and all were utilized before their expiration date. The dilutions of essential oils were prepared immediately in advance of the experiments.

### 2.3. Chemicals

Nutrient broth and TSB (Tryptic Soy Broth) medium were purchased from Oxoid Ltd. (Basingstoke, UK); Mueller-Hinton medium from Biocorp Polska Sp. z o.o. (Warsaw, Poland); DMSO (dimethyl sulfoxide) and PBS buffers (phosphate-buffered saline) from POCH S.A. (Gliwice, Poland); and RedoxSensor^TM^ Green as well as propidium iodide from Thermo Fisher Scientific (Basingstoke, UK). 

### 2.4. Estimation of Bioactive Compounds Content in Investigated Essential Oils by Gas Chromatography

Gas chromatography was carried out on an Agilent Model 7890A (Agilent Technologies, Santa Clara, CA, USA), with a flame ionization detector equipped with an automatic sample tray [21]. The HP-5 column (Agilent Technologies, Santa Clara, CA, USA) 30 cm long and 0.32 mm inner diameter was used. The thickness of the film of the stationary layer was 0.25 μm. The flow rate of the carrier gas (nitrogen) was 2.15 mL/min. The temperatures of the injector and detector were 280 and 300 °C, respectively. The column temperature was programmed from 50 to 300 °C at 5 °C/min. For standard calibration curves, the ratio of the peak height of bioactive compounds to that of the internal standard substances was plotted as a ratio of the amount of the former to a definite amount of the latter (Agilent ChemStation Software, Santa Clara, CA, USA).

### 2.5. Agar Disk Diffusion Assay

A very preliminary estimation of the inhibitory effect of the investigated essential oils (bergamot, nutmeg, bitter orange, lime, lemongrass, juniper, black pepper, rosemary, and St. John’s wort) on the two *P. orientalis* strains was determined by agar disk diffusion method by Bauer et al. [22]. Precisely 0.2 mL of bacterial suspension (10^6^ CFU/mL) in TSB medium was streaked on Petri dishes containing Mueller-Hinton medium. Sterile filter paper disks of 5 mm diameter were soaked with 2 μL of each of the essential oils and placed on the surface of the dishes. The erythromycin (Oxoid Ltd., Basingstoke, UK) and DMSO 0.5% (vol/vol) in sterile deionized water were used as positive and negative controls, respectively. The plates were incubated at 36 °C for 24 h aerobically. After incubation, the diameter of the inhibition zones was measured in millimeters. The diameter of the clear zone (after deducting the diameter of the disk) was the result. The inhibition zone with a diameter >2 mm was considered positive. Each assay was performed in triplicate in three separate experimental runs.

### 2.6. Determination of Minimum Inhibitory Concentration (MIC) and Minimum Bactericidal Concentration (MBC)

A determination of MIC values was performed according to the method described by Weerakkody et al. [23], with some modifications. The essential oils were diluted in series. Zero point two milliliters (0.2 mL) of oil was introduced into the first tube containing 2 mL of the Mueller-Hinton medium. After mixing from the tube, 200 μL of liquid was removed and transferred to another tube. This operation was repeated four times. Each tube containing the serial dilutions of one of the tested essential oils (bergamot, nutmeg, bitter orange, lime, lemongrass, juniper, black pepper, rosemary, and St. John’s wort) and separately bioactive compounds (linalool and citral) was inoculated by 0.2 mL of bacterial suspension with a turbidity of 1 McFarland (app. 10^6^ CFU/mL). The control probe, i.e., without essential oils, was a test tube containing 2 mL of Mueller-Hinton broth and 0.2 mL of the bacterial suspension. All probes were incubated aerobically (36 °C, 48 h). The MIC value was determined by the lowest concentration of the essential oil at which no bacterial growth was observed (clouding of the medium). Additionally, the probes in which no bacterial growth was observed were subjected to spectrophotometric analysis. Spectrophotometric measurement was carried out at a wavelength of 580 nm. The results were compared with the absorbance of the control probes, and the percentage inhibition of the bacteria growth was calculated. The essential oils that did not show antimicrobial activity against the tested strains were eliminated from further analysis. 

In addition, the growth curve of the bacteria incubated with selected essentials oil in MIC value was determined and compared with the growth curves of the cells cultivated without essential oils. The dynamics of the bacterial growth were evaluated by log of cell number versus time.

To determine the MBC value, those dilutions of oils that showed antimicrobial activity on the tested strains were selected. For this, 1 mL of the suspension from the proper probes (determined as MIC) was spread on Mueller-Hinton Agar Petri dishes and incubated aerobically (36 °C, 24 h and 15 °C, 72 h). The MBC values were determined as the concentration of the essential oils which inhibited bacteria growth by 99% relative to the control probe. Each assay was performed in triplicate in three separate experimental runs.

### 2.7. Flow Cytometry Analysis

The BD FACS Aria Aria^TM^III (Becton Dickinson, Franklin Lake, NJ, USA) flow cytometer (cell sorter), equipped with 4 lasers (375, 405, 488, and 633 nm) and 11 fluorescence detectors, both forward scatter (FSC) and side scatter (SSC) detectors, was used [24]. The redox potential determines the internal metabolic activity of cells. Thus, the redox potential in the bacterial cells cultivated with the essential oils was compared with the redox potential in bacterial cells cultivated without the oils. Flow cytometric analysis of microbial cells’ vitality and metabolic activity, with redox potential as a relevant parameter, were evaluated using two non-fluorescent parameters, Forward Scatter (FSC) and Side Scatter (SSC), and two fluorescent parameters, green fluorescent (detector FL1) from BacLight Redox Sensor Green Vitality Kit reagent using 530/30 band pass filter and red fluorescent (detector FL2) from propidium iodide reagent using 616/23 band pass filter. For excitation of both the fluorescent reagents, a 488 nm laser was employed. Flow cytometry analyses were performed using logarithmic gains and the specific detectors’ settings. The threshold was set on the FSC signal. Data was acquired in a four-decade logarithmic scale as area signals (FSC-A, SSC-A, FL1-A and FL2-A) and analyzed with FACS DIVA software (Becton Dickinson). The analysis of the fluorescence signals from both fluorochromes preceded doublets discrimination procedure, using height versus width scatter signals measurement, in order to discriminate single events from conglomerates. The populations were then defined by gating in the dot plots of green fluorescence (FL1) versus red fluorescence (FL2). Each sample was analyzed in triplicate. Estimation of the cells’ redox potential was performed using medians of green fluorescence (FL1) signals of the gated populations as defined on the bivariate dot plot (FL1 vs. FL2).

For flow cytometry analyses, 1 mL of nutrient medium supplemented by individual essential oils in MIC concentration was inoculated with *P. orientalis* (10% vol/vol) and incubated (36 °C, 24 h and 15 °C, 72 h). For analysis, each culture was collected by centrifugation, re-suspended in 1% PBS and prepared according to the manufacturer’s manual. Prior to analysis, an optimization step was employed in order to assess the reagents’ appropriate staining concentrations.

### 2.8. Observations of Morphological Changes in Bacterial Cells

The observations of the morphological changes in the bacterial cells were carried out in microscopic preparations colored with crystal violet. Bacterial cells (10% vol/vol inoculum) were incubated (15 °C, 72 h) in a DMSO buffer supplemented with the essential oils in MIC concentrations. The control probe was prepared from the DMSO buffer and bacteria inoculum (but without the essential oil). Bacterial preparations were observed using the transillumination mode of the inverted microscope (Zeiss Axiovert 200, Oberkochen, Germany) with 100× magnification of the objective. Measurement of the size (length and width) of the cells was made using AxioVision computer software (Zeiss, Germany). The experiment was performed in duplicate.

### 2.9. Evaluation of Membrane Integrity

The integrity of the cell membrane was evaluated by measuring the release of cell proteins into the cell suspension as a result of the essential oil action. According to the methods used by Lv et al. [25], bacterial cells from the suspension (10 mL) of both *P. orientalis* strains were collected by centrifugation for 10 min at 3000 g. Cells were washed three times and re-suspended in 10 mL of PBS (0.1 M, pH 7.4). Then re-suspended cells were shacked and incubated at 36 °C for 24 h in the presence of the individual essential oils in MIC concentrations. The suspensions were then centrifuged at 6000 g for 5 min. Next, 0.1 mL of the supernatants was diluted with 5 mL of Bradford reagent (Pol-Aura, Różnowo, Poland) and incubated at room temperature (30 min), and the absorption at 595 nm was measured by using an UV–vis spectrophotometer. Correction was carried out for absorption of the suspension with PBS (0.1 M, pH 7.4) containing the same concentration of the essential oil directly after the addition of that oil. The concentration of the proteins in suspension was carried out following Bradford’s method [26]. An untreated sample was used as a control. The standard curve of absorbance (A) from the concentration of protein (mg/mL) (B) is expressed by the equation: A = 0.985 × B.

### 2.10. Statistical Analysis 

All experiments were done in duplicate or triplicate, and mean values are presented. The statistical tests were performed with a significance level of α = 0.05 using Statistica 13 software (Dell Software Inc., Aliso Viejo, CA, USA).

## 3. Results and Discussion

### 3.1. Effect of Essential Oils on Bacterial Growth

The disc diffusion method was used as a very preliminary method to recognize which essential oils demonstrated antibacterial action against the investigated *P. orientalis* strains. This method is very simple but the results require confirmation by more reliable methods. The obtained results depend, inter alia, on the thickness of the substrate layer, the arrangement of the paper discs, the concentration of the substance in the discs, and the density of the inoculum, etc. In addition, the investigated substances may have different agar diffusion capacities, which may bring about an overestimation or underestimation of the result obtained [27]. Thus, in our experiments, we additionally calculated the MIC value of the individual essential oils. As can be seen in Table 1, MIC values do not always correlate with the size of the clear zone (e.g., lime and black pepper in the case of *P. orientalis* P110). The largest zones of inhibition of *P. orientalis* P110 growth were observed in the presence of lime (19 mm) and lemongrass (18 mm) oils, while the greatest inhibition of *P. oriantalis* P49 occurred with lemongrass and bitter orange oils (11 and 9 mm, respectively). In the literature, there is no information whatsoever regarding the antibacterial action of essential oils on *P. orientalis* except the work published by the authors concerning the effect of oils on the metabolic and physiological properties of *P. orientalis* bacteria [21]. However, it should be emphasized that, in that work, commercial oils were used and the oils were independently extracted. For this reason, these results cannot be compared. This observation, however, led the authors to further studies in which the effectiveness of the antimicrobial action of both types of oils was compared. In the literature, no information can be found on the inhibition of the growth of *P. aeruginosa* and *P. fluorescens* by lemongrass and lime oils [28]. Another work by Ghabraie et al. [29] demonstrated that juniper and rosemary oils have no antibacterial action on *Pseudomonas* spp. (in the disc diffusion method which—in the opinion of the present authors—is not objective). However, this is not in line with the observations of Prabuseenivasan et al. [28], in which it was stated that rosemary oil inhibits the growth of *P. aeruginosa* (the inhibition zone was equal to 23.4 mm). Furthermore, Alizadeh et al. [30] documented that rosemary oil exhibits antibacterial action against *P. fluorescens* (the inhibition zone equals 15.1 mm).

We analyzed the percentage content of bioactive compounds in tested plant oils. The results are presented in Table 2. The tested oils significantly differ from one another in the ratio of individual bioactive components. Cineol, *p*-cymene and camphor were detected only in rosemary oil. According to the literature data, the main components of rosemary oil are cineole and camphor; this is in line with our observations. Rosemary oil is widely used for flavoring and as a natural preservative in foods. It is a rich source of antioxidants. The antioxidant traits of rosemary oil have been attributed to the presence of phenolic diterpenes that scavenge hydroxyl radicals, singlet oxygen, and lipid peroxyl radicals [31]. Eugenol, myristicin, α-thujene, β-bisbolene, and geranyl acetate were observed only in nutmeg. According to literature data, the volatile components represented 99.3% of the total in nutmeg oil. The percentage of major and most important constituents are α-terpinenol (31.3%—in our oil only 5%), γ-terpinene (7.8%—in our oil 4.4%), myristicin (7.1%—in our oil 9.5%), and *p*-cymene (6.5%—not present in our oil) [32]. The method of obtaining oils was the same as in our research, but the methods of plant breeding, harvesting time, etc. exert a significant influence on the bioactive composition of the oils. We observed that the main components of nutmeg oil were sabinene (18%), α-pinene (17.5%) and β-pinene (14.5%). While the presence of α-pinene and β-pinene was confirmed in all oils except α-pinene in bergamot and lemongrass and β-pinene in lemongrass. Nbiha et al. [33] observed that bergamot oil, obtained using a hydrodistillation method, had both α-pinene and β-pinene present in concentrations of 0.48% and 4.38% respectively. The main components of that oil are limonene (59.21%—in our oil 34.5%), linalyl acetate (16.82%—not detected in our oil), and linalool (9.52%—in our oil 12%). Among the main bioactive components of lemongrass are citral (68%, in literature 78.6%), geranial (37%, in the literature 48.1%) and neral (30%, in the literature 34.6%). Bassolé et al. [34] also noted the presence of myrcene (11%), but this was not detected in our investigations. Furthermore, we estimated the MIC value of two bioactive compounds, linalool and citral, because the literature regarding the antibacterial activities of citral and linalool on *Pseudomonas* ssp. are contradictory [35,36]. What is important is that both citral and linalool are used in large quantities in food processing (because, among other factors, of their high aroma values) [37]. Moreover, citral was observed only in lemongrass oil and has the largest clear zone of all tested oils. Surprisingly, our research showed that citral has no inhibitory effect on *P. orientalis* P49 and P110, and linalool inhibits only *P. orientalis* P110. Dorman and Deans [38] stated that citral inhibits the growth of *P. aeruginosa* but linalool does not. Thus, we can suppose that the antibacterial force of such oils is mostly dependent on the interaction of the many bioactive components (i.e., a synergistic effect—target of our future study). 

With the exception of the authors’ own work describing the influence of selected essential oils on the physical and biochemical properties of *P. orientalis* bacteria [21], the literature has no data regarding essential oils’ inhibitory action on *P. orientalis* growth. Consequently, no research on MIC values has been described by other scientists. Our investigations have shown that lemongrass and black pepper oils inhibit the growth of *P. orientalis* P110 in the lowest concentrations in comparison with the other tested oils. The degree of inhibition of bacterial growth was equal to 62.7% and 64.5% for lemongrass and black pepper respectively. The result for black pepper was quite surprising due to the lack of a zone of growth inhibition in the disc test. It is known, however, that this method is fraught with errors, including the fact that the obtained result is influenced by, among other factors, the thickness of the substrate layer, the arrangement of the paper discs, and the concentration of the substances in the discs. It should be noted that test substances may have different abilities to diffuse in agar. This may cause an overestimation or underestimation of the obtained result, and therefore these studies require confirmation by other methods, such as by determining the minimum concentrations that inhibit bacterial growth [39]. The MIC analysis indicated that bergamot and bitter orange oils in the same concentrations (8.3 μL/mL) as black pepper oil are able to inhibit the growth of *P. orientalis* P49. The levels of inhibition of bacterial growth were lower than for *P. orientalis* P110—33.6% and 62.7%, respectively. It is difficult and unreliable to compare these results with those of other *Pseudomonas* representatives. The results are often given in different volume units of used oils (%, mg/mL, mg/mol, etc.). In addition, the source and the method of obtaining the oils have a significant impact on the result. For example, Hammer et al. [40] stated that lemongrass oil inhibits *P. aeruginosa* in concentrations of 1% (vol/vol). Naik et al. [41], in turn, stated that lemongrass is not able to inhibit *P. aeruginosa* even in higher concentrations. Lime oil in our experiment inhibits the growth of *P. orientalis* P110 only in concentrations of 90.9 μL/mL (the proportion in % of the inhibition of growth was, however, high, equal to 85%). According to Prabuseenivasan et al. [28], lime oil is able to inhibit the *P. aeruginosa* growth in concentrations of 6.4 mg/mL. *P. orientalis* growth was inhibited by rosemary and juniper oils in concentrations of 90.9 μL/mL (except for the *P. orientalis* P49 strain, the growth of which was not inhibited by rosemary). On the other hand, Ghabraie et al. [29] demonstrated that rosemary and lemongrass oils inhibited the growth of *P. aeruginosa* only in high concentrations. Alizadeh et al. [30] also confirmed the antibacterial activity of rosemary oil (in concentrations of 3.0 mg/mL) against *P. fluorescens*. Citral did not inhibit *P. orientalis* in the investigated concentrations. Linalool inhibited less than 50% of *P. orientalis* P110 cells in concentrations equal to 90.9 μL/mL. Cox and Markham [35] stated that linalool inhibited the growth of *P. aeruginosa* in concentrations of 1.72 mg/100 mL and in concentrations of 1.78 mg/100 mL for citral.

In conclusion, there is no correlation between MICs for *P. orientalis* and other bacteria of the genus *Pseudomonas*. Moreover, significant differentiation can be observed in the sensitivity of *P. orientalis* strains to the tested oils. This indicates a very high biodiversity in the strains (especially those isolated from environmental probes). Therefore, it is necessary to develop the MIC values of the specific oils for each strain individually. However, the abovementioned results indicated that bergamot, bitter orange, lemongrass, lime, black pepper, juniper, and rosemary oils could be successfully used as natural preservatives for fish products. Moreover, we have started sensory studies on fish prepared in a rapeseed oil with selected essential oils. Preliminary analyses indicate that bergamot, bitter orange, lemongrass, and lime oils are better rated than black pepper, juniper, and rosemary oils. In the next stage of the study, we investigate the synergistic antimicrobial effect of the oils, which, in our study, showed the highest antimicrobial activity.

Of all of the tested oils, none showed bactericidal activity (in the tested concentrations). The bactericidal effect was observed only in the case of a bioactive compound—linalool—against *P. orientalis* P110 (at a concentration already corresponding to the MIC value). However, it is worth emphasizing that linalool, in pure form, occurs in oils in a quantity smaller than the MIC value determined in our experiments. The bacteriostatic action of oils in low concentrations is their advantage as such low concentrations do not cause the formation of resistance mechanisms in microorganisms. In addition, their use in foods in order to inhibit the growth of saprophytic bacteria is ideal already at minimal concentrations because higher concentrations affect the sensory characteristics of the food products [1]. Essential oils, which showed no antimicrobial or antibacterial properties in relation to each particular strain of *P. orientalis*, were not subjected to further studies. In the case of *P. orientalis* P49, nutmeg, lime, rosemary and St. John’s wort oils were excluded, whereas bergamot, nutmeg, bitter orange and St. John’s wort oils were excluded for *P. orientalis* P110.

### 3.2. The Growth Curve of P. Orientalis P110

**Figure 1:**
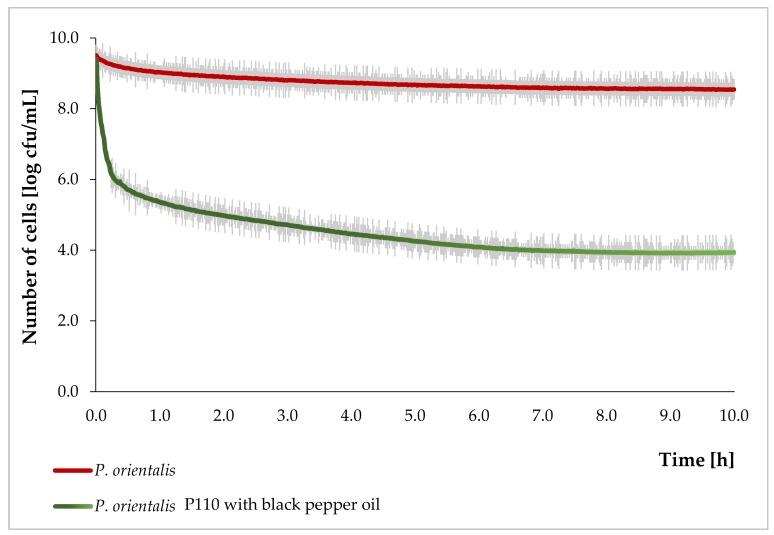
*P. orientalis* P110 growth curve with and without pepper essential oil.

In order to confirm the inhibition of *P. orientalis* bacterial growth by the essential oils, the bacterial growth curve was determined (Figure 1 presents the results—the normal growth curve and growth curve for bacteria incubated with black pepper). The control probe was a culture without oil, while the tested one was with oil at the MIC concentration. The *P. orientalis* P110 strain and pepper oil were deliberately chosen because discrepancies were observed in the results of the disc-diffusion method and the serial dilutions. At the start of the measurement, bacteria were in a stationary phase. The addition of oil caused a rapid decline in the population of bacteria, amounting to almost four logarithmic cycles in the 1st h of cultivation. In the following hours, the decline in bacterial count was significantly lower until a constant value was obtained after 7 h of measurement. After 10 h, the difference in the number of bacteria in culture without and with oil was about five log cycles. Based on these observations, it can be concluded that black pepper oil penetrates the cells of the *P. orientalis* within the first 30 min after application, causing a rapid decrease in the population of bacteria in the 1st h.

### 3.3. Investigation of Metabolic Activity of Cells Using a Flow Cytometer

Using a flow cytometer, the intracellular oxidoreduction potential of *P. orientalis* bacteria was tested in a culture without the addition of essential oils and once with the addition of oils in MIC concentrations (Table 3). The cells were divided into three metabolic fractions—demonstrating high, medium and low metabolic activity. This analysis allows changes in the metabolic activity of cells under the influence of the oils to be demonstrated.

The metabolic activity of *P. orientalis* P49 cells was mostly weakened under the influence of bergamot oil. Lemongrass oil also limited the intracellular metabolic activity of *P. orientalis* P49 cells. In the case of *P. orientalis* P110, rosemary and lemongrass oils in MIC concentrations caused a significant decrease in metabolic activity of the culture compared with the control probe (cells with the lowest metabolic activity were the largest group). Black pepper and juniper oils did not significantly influence the intracellular activity of *P. orientalis* P110 cells. 

**Figure 2:**
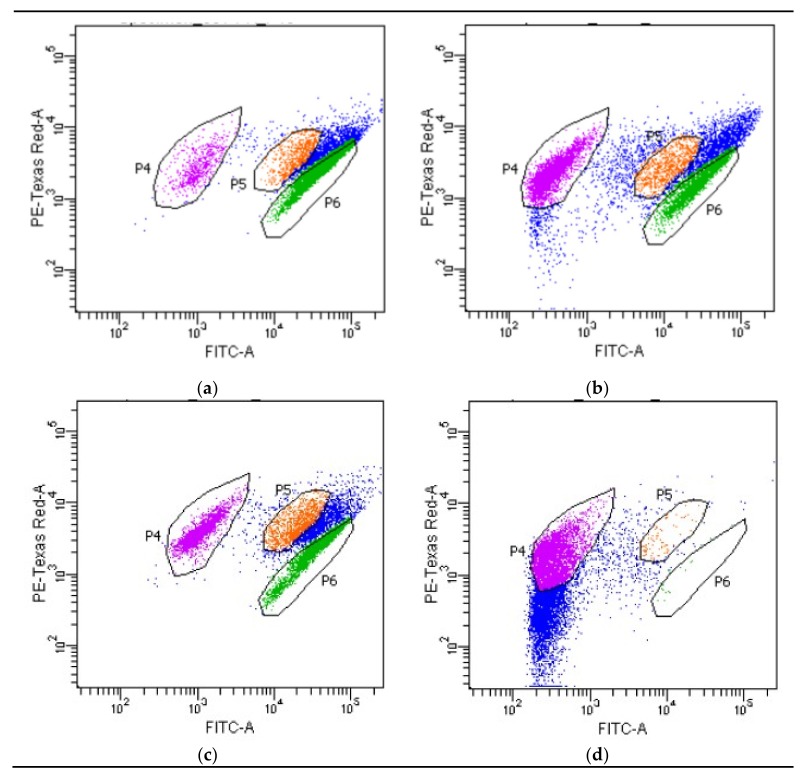
The distribution of individual cell fractions (36 °C, 24 h): (**a**) control probe P49; (**b**) *P. orientalis* P49 incubated with bergamot oil; (**c**) control probe P110; (**d**) *P. orientalis* P110 incubated with rosemary oil.

Figure 2 shows the distribution of individual cell fractions, using the example of the *P. orientalis* P49 strain. The graph for bergamot oil has been chosen because bergamot oil caused the largest decrease in the number (proportion) of cells with high metabolic activity. The cells with the higher metabolic activity are the green fraction (P6), the orange fraction (P5) includes the cells with medium intracellular activity, and the violet one is a fraction of the lower activity cells.

Fluorescence-based flow cytometric methods are very useful, sensitive alternative analytical tools in microbiology. They provide accurate and rapid analyses of the viability and vitality of single cells with high levels of sensitivity [42]. Accordingly, we can more credibly assess the effect of the oils on living cells. Flow cytometry was used also by other researchers to estimate the viability of bacterial cells incubated with some essential oils. For example, Nguefack et al. [42] investigated the antibacterial activity of essential oils from *Cymbopogon citratus*, *Ocimum gratissimum* and *Thymus vulgaris* against *Listeria innocua*. Additionally, other researchers have compared the effectiveness of two methods, agar well diffusion and flow cytometry, to determine the antibacterial effect. The results obtained from both methods were not always in accordance. This is another proof of the unreliability of the disc method. Silva et al. [43] investigated the antibacterial action of coriander essential oil on 12 strains of bacteria (both Gram-positive and Gram-negative), including *P. aeruginosa*. The results were presented as a percentage cells’ viability reduction. Paparella et al. [44] applied flow cytometry to assess the antimicrobial activity of oregano, thyme and cinnamon essential oils on *Listeria monocytogenes*. The authors drew a very important conclusion from their research. They observed significant differences between plate count results and flow cytometric data, which suggested the presence of a sub-lethally stressed subpopulation not able to form colonies on agar plates. Thus, the use of an alternative method (not only cultivation) is important in such research. The influence of some natural chemicals (oregano, rosemary and laurel extracts) on the growth and viability of *Listeria monocytogenes* by flow cytometry technique was also investigated by Muñoz et al. [45]. Thus, bacteria cells were divided into three different cell populations, specifically: living, dead, and compromised cells. Additionally, live cell percentage decreased with time of exposure, whereas the percentage of compromised cells remained constant and dead cells increased during the same period. From our point of view, the results obtained from that analysis are very important. The cells were divided into three sections—with high, medium and low metabolic activity. It is an additional proof that essential oils in low concentrations do not cause the death of bacterial cells. Moreover, flow cytometry indicated the existence of viable but nonculturable (VBNC) bacteria which means that bacteria in a state of very low metabolic activity do not divide, but are alive and have the ability to become culturable once resuscitated. Bacteria in a VBNC state cannot grow on standard growth media. Bacteria can enter the VBNC state as a response to stress (contact with essential oils) [46].

### 3.4. Influence of the Temperature and Time Incubation on the Efficiency of Essential Oil Antibacterial Action

*P. orientalis* are able to grow at reduced temperatures. Moreover, they are saprophytic bacteria that spoil, among others, fish products. Food products, as a rule, should be stored at low temperatures. Moreover, it is important that products remain in good quality (including microbiological safety) for a prolonged period of time. Thus, we decided to investigate the antibacterial activity of essential oils with proven effect on both tested *P. orientalis* strains during incubation in 15 °C through 72 h. The results of flow cytometry analysis are presented in Table 3. We obtained three fractions of cells (with low, medium, and high intracellular activity). However, the results were a surprise for us. It appeared that, including lemongrass, the oils in MIC values determined at 36 °C did not significantly influence metabolic activity. The percentage of viable cells was comparable in cultivations with oils to the control probe without oils. We concluded that temperature plays a very important role in the antimicrobial activity of essential oils. That is why we again set the MIC value at a reduced temperature (15 °C, 72 h) (Table 3).

In all our investigated oils, MIC values were significantly higher than ones determined at 36 °C. The MIC values for lime and rosemary oils (*P. orientalis* P110 strain) were almost twice as high as at the temperature of 36 °C. For lemongrass, it was 20 times higher. The applied concentrations of pepper and juniper oils did not inhibit the growth of *P. orientalis* P110 (MIC > 166.7 μL/mL). In the case of *P. orientalis* P49, the MIC values for lemongrass, bergamot and bitter orange oils obtained at 15 °C were 11 times higher than at 36 °C. Interestingly, the MIC values at 15 °C were significantly higher for the *P. orientalis* P110 strain than for *P. orientalis* P49. At the same time, the ratio of bacterial growth inhibition was much lower in the P110 strain than for P49. The flow cytometry analyses were carried out again. The percentages obtained of all three fractions of bacteria were very similar to the results obtained at 36 °C (±2.5%) (Table 2). 

The reduced efficiency of antibacterial activity of essential oils at lower temperatures is associated with a limited rate of diffusion of essential oils to bacterial cells in such lower temperatures, as also demonstrated by Wojtyś and Jankowski [47]. The rate of orange and peppermint oils penetration into bacterial cells was strictly dependent on the incubation temperature (the higher temperature, the higher penetration rate). The Van’t Hoff rule says that a drop in temperature by 10 °C causes a two- to four-fold reduction in the speed of chemical reactions. However, essential oils are complex mixtures of many bioactive components between which synergistic interaction takes place [48]. Thus, even a small change in temperature causes significant changes in the efficiency of their action.

To conclude, it is thus possible to inhibit the growth of *P. orientalis* bacteria using low temperatures. However, to do so, the doses of oils must be significantly higher. Therefore, before their application to a food product, it is necessary to perform cyto- and genotoxicity analyses in the MIC values determined at 15 °C.

### 3.5. The Mode of Action of Essential Oils

Essential oils are active against a variety of targets, particularly the membrane and cytoplasm, and in some cases, they are able to change the morphology of the cell [9]. The activity of individual essential oils differs depending on, inter alia, the shape of the bacteria. The rod-shaped bacterial cells have been reported to be more sensitive to plant oils than coccoid cells [9,49]. 

*P. orientalis* are rod-shaped bacteria. The *P. orientalis* P49 cells reach an average of 1.12 μm length and 0.55 μm width; *P. orientalis* P110 cells are 1.02 μm long and 0.49 μm wide. These are comparable with other bacteria from *Pseudomonas* genera. *P. aeruginosa* cells are 1.50 μm long and 0.80 μm wide; *P. fluorescens* cells are 1.50 μm long and 0.51 μm wide; and *P. syringa* cells are 1–2 μm long and 0.80 μm wide [50].

**Figure 3:**
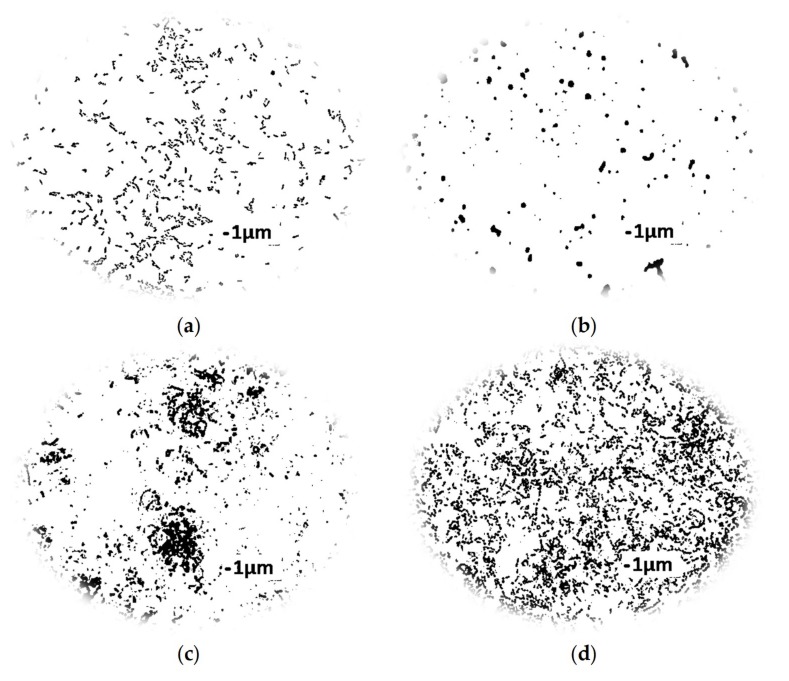

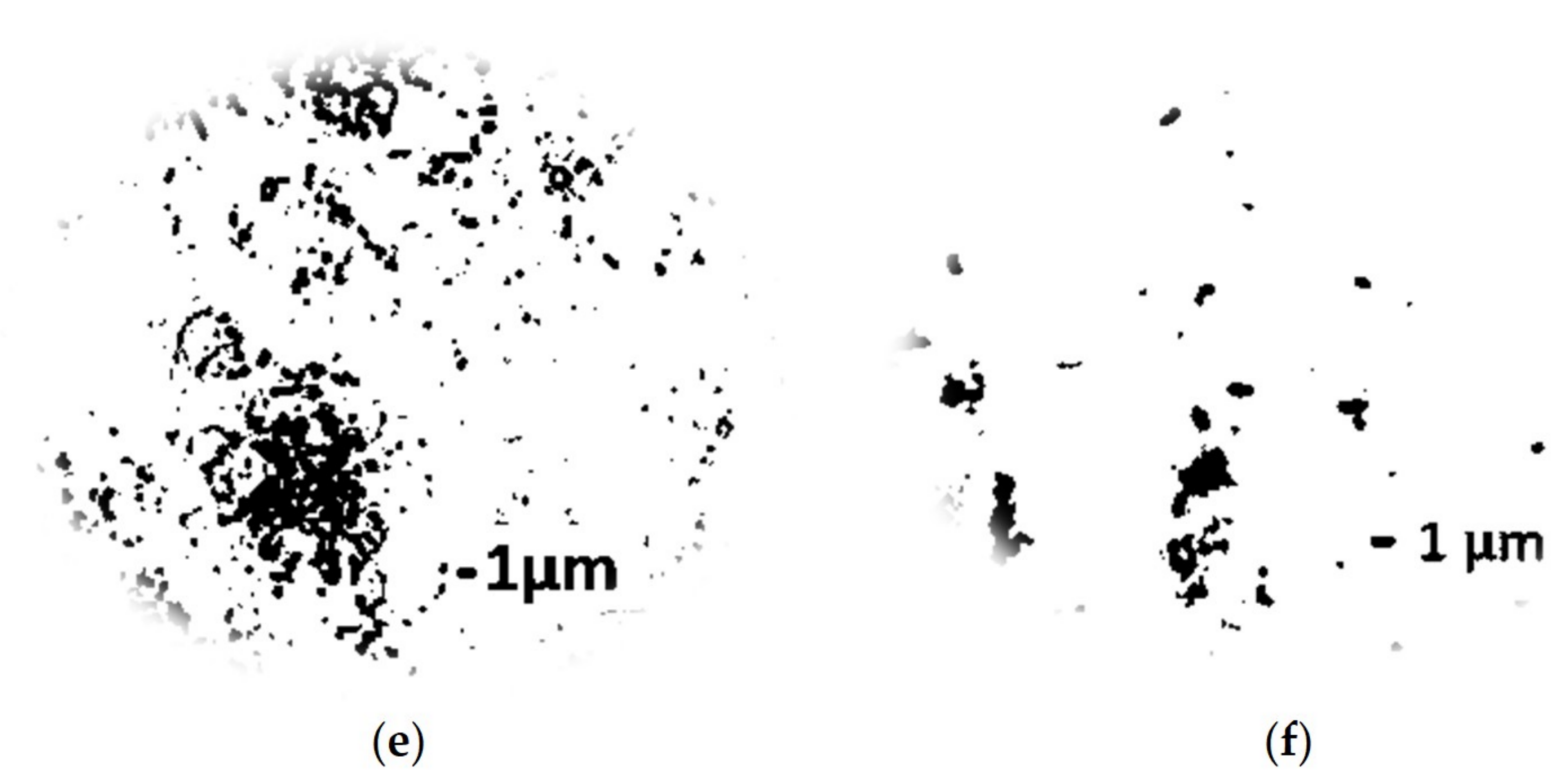
The cell morphology: (**a**) control probe P49; (**b**) *P. orientalis* P49 incubated with juniper; (**c**) *P. orientalis* P49 incubated with bitter orange; (**d**) control probe P110; (**e**) *P. orientalis* P110 incubated with black pepper; (**f**) *P. orientalis* P110 incubated with lemongrass oil.

In the case of both investigated *P. orientalis* strains, after incubation with essential oil (in MIC values), some significant changes in bacteria cells’ shape were observed. Under the influence of essential oils, cells became coccoid. In all probes, the length and width of the cell were very similar to each other. Also, depending on applied oil, the cells’ sizes differ. In some cases, they were conglomerated (e.g., after incubation of cells with lemongrass). The essential oils disturb the proper structure of the cell membrane and, as a consequence, the cytoplasm (and intracellular constituents) can leave the cell, thereby affecting its size and causing alterations to the outer envelope [9]. In general, *P. orientalis* P49 cells were smaller than *P. orientalis* P110. These changes in cell shapes are demonstrated in Figure 3.

Because the changes in the cells’ shapes are directly connected with alterations to the membrane protein, we decided to estimate the concentrations of protein released outside the cell (cell-free supernatants after treatment with the essential oil components were analyzed). Generally, bacteria from *Pseudomonas* genera are naturally able to produce a large quantity of exopolysaccharides (EPS—extracellular polymeric substances) including proteins and polysaccharides. These substances are synthetized in order to protect cells from adverse physical and chemical factors (such as the lack of water, rapid temperature changes and disinfectants) [51]. Thus, in the first step, it was necessary to estimate the concentration of extracellular proteins in a control probe (without incubation with essential oils). Results obtained in probes incubated with plant oils were compared with the results of a blank probe. The analyses were carried out for both the investigated *P. orientalis* strains. In the case of *P. orientalis* P49, the influences of bergamot, bitter orange, and lemongrass on the integrity of the cell membrane were investigated. *P. orientalis* P110 bacteria were tested after incubation with lime, black pepper, and lemongrass. The concentrations of extracellular proteins in control probes were comparable for both strains. However, in probes incubated with selected oils, the increase in the concentration of proteins was observed in all cases. Additionally, it was clear that the concentrations of proteins differed in each of the individual oils. In a control probe (for both strains), the protein concentration was 0.5 mg/mL. The highest concentrations of proteins were observed in probes with lemongrass (0.12 mg/mL) for *P. orientalis* P49 strain, and for lime (0.11 mg/mL) for *P. orientalis* P110. In probes incubated with bergamot and bitter orange, the protein concentration was equal to 0.9 mg/mL for the *P. orientalis* P49 strain. The same protein concentration was observed for lemongrass and black pepper in *P. orientalis* P110. A comparable observation was described in the work of Nazzaro et al. [9]—the authors stated that essential oils and their individual components can act, to a different degree, on proteins present in bacteria and may affect their outflow from the cells. The lemongrass oil (in case of P49 strain) and lime oil (in case of P110 strain) caused an almost two-fold increase in the concentration of extracellular protein. This proves the effective action of these oils on inhibiting the activity on the tested strains. These results are also confirmed by the results of flow cytometry: In both cases, the total percentage of cells in the low and medium activity fractions exceeded the percentage of the cells in the high activity fraction. Additionally, significant changes in the cells incubated with these oils were observed (the cells change to a coccoid shape). 

The release of bacterial membrane protein as a result of essential oil antibacterial action was also investigated by other authors. For example, Devi et al. [52] described the influence of eugenol on membrane proteins in *Salmonella typhi* cells. Eugenol caused an increase in the concentration of proteins in a post-culture medium and, as a consequence, damaged the cytoplasmic membrane and caused subsequent leakage of intracellular constituents. Wang et al. [53] tested the influence of cinnamon bark essential oil on the integrity of *Porphyromonas gingivalis* cell membranes. Protein leakage was observed when cinnamon oil was added into a cultivation medium. Thus, membrane integrity was disturbed by enhancing the cell permeability, and several morphological changes in *P. gingivalis* cells were also observed.

## 4. Conclusions

Our research indicated highly varied antibacterial activity in a series of investigated essential oils on *P. orientalis* P49 and P110 strains. Moreover, a diversified sensitivity of the tested strains to individual oils was observed, thereby indicating high biodiversity among the representatives of the *P. orientalis* species. Essential oils have multiple modes of action—they change the shape of the cell, and this is directly associated with the interaction of the oils on the cell membrane and the release of membrane proteins. Essential oils are also able to weaken the metabolic activity of cells without causing cell death. The great advantage of this mechanism is that it does not induce, in the cell, bacterial mechanisms of resistance to the acting agent. Our research has shown that essential oils and their concentrations should be selected individually for each strain. In addition, breeding conditions are very important—temperature has a big impact on the effectiveness of the antimicrobial effect of essential oils. In further experiments, we will carry out analyses to compare the antimicrobial activity of commercial oils with oils obtained in our laboratory. Moreover, we will examine the degree of inhibition of bacterial growth in a food product model and conduct sensory tests of mixtures of oils in fish products.

## Figures and Tables

**Table 1:** The effect of agar disk diffusion method and MIC values of the investigated essential oils (36 °C, 24 h), and the MIC values of essential oils (15 °C, 72 h).

Essential Oil/Bioactive Compounds	Bacteria Strain	Zone of Growth Inhibition [mm]	MIC Value [μL/mL] 36 °C, 24 h	MIC Value [μL/mL] 15 °C, 72 h	MIC Value [mg/mL] 36 °C, 24 h	MIC Value [mg/mL] 15 °C, 72 h	The Degree of Inhibition of Bacterial Growth [%] 36 °C, 24 h	The Degree of Inhibition of Bacterial Growth [%] 15 °C, 72 h
Bergamot	*P. orientalis* P49	5.0 ^c^ ± 0.6	8.3 ^a^ ± 0.4	90.9 ^a^ ± 2.7	7.5 ^a^ ± 0.5	82.5 ^a^ ± 3.1	33.6 ^b^ ± 2.1	41.9 ^d^ ± 2.6
	*P. orientalis* P110	0 ^a^	-	-	-	-	0 ^a^	0 ^a^
Nutmeg	*P. orientalis* P49	0 ^a^	-	-	-	-	0 ^a^	0 ^a^
	*P. orientalis* P110	0 ^a^	-	-	-	-	0 ^a^	0 ^a^
bitter orange	*P. orientalis* P49	9.1 ^d^ ± 0.9	8.3 ^a^ ± 0.3	90.9 ^a^ ± 4.6	6.9 ^a^ ± 0.2	76.3 ^a^ ± 2.2	62.7 ^c^ ± 1.4	2.7 ^b^ ± 1.5
	*P. orientalis* P110	0 ^a^	-	-	-	-	0 ^a^	0 ^a^
Lime	*P. orientalis* P49	0 ^a^	-	-	-	-	0 ^a^	0 ^a^
	*P. orientalis* P110	19.2 ^d^ ± 1.0	90.9 ^b^ ± 7.6	166.7 ^a^ ± 8.9	81.5 ^b^ ± 6.5	149.5 ^a^ ± 10.2	85.0 ^d^ ± 0.2	25.7 ^c^ ± 2.4
Lemongrass	*P. orientalis* P49	11.1 ^d^ ± 1.1	8.3 ^a^ ± 0.4	90.9 ^a^ ± 8.3	7.6 ^a^ ± 0.2	83.7 ^a^ ± 7.6	48.5 ^c^ ± 1.8	30.3 ^c^ ± 3.1
	*P. orientalis* P110	18.1 ^d^ ± 1.9	8.3 ^a^ ± 0.6	166.7 ^a^ ± 11.2	7.6 ^a^ ± 0.3	153.5 ^a^ ± 9.8	62.7 ^c^ ± 5.7	13.4 ^b^ ± 4.5
Juniper	*P. orientalis* P49	2.0 ^b^ ± 0.3	90.9 ^b^ ± 5.1	-	76.0 ^b^ ± 8.1	-	40.7 ^c^ ± 2.5	0 ^a^
	*P. orientalis* P110	2.1 ^b^ ± 0.4	90.9 ^b^ ± 4.3	-	76.0 ^b^ ± 9.3	-	54.9 ^c^ ± 1.5	0 ^a^
black pepper	*P. orientalis* P49	2.0 ^b^ ± 0.2	90.9 ^b^ ± 5.9	-	78.7 ^b^ ± 2.6	-	49.9 ^c^ ± 0.7	0 ^a^
	*P. orientalis* P110	0 ^a^	8.3 ^a^ ± 0.2	-	7.2 ^a^ ± 0.3	-	64.5 ^c^ ± 2.9	0 ^a^
Rosemary	*P. orientalis* P49	0 ^a^	-	-	-	-	0 ^a^	0 ^a^
	*P. orientalis* P110	7.0 ^c^ ± 1.0	90.9 ^b^ ± 7.1	166.7 ^a^ ± 14.2	80.1 ^b^ ± 6.3	146.9 ^a^ ± 10.8	64.5 ^c^ ± 2.6	11.3 ^b^ ± 3.7
St. John’s wort	*P. orientalis* P49	2.0 ^b^ ± 0.3	-	-	-	-	0 ^a^	0 ^a^
	*P. orientalis* P110	2.0 ^b^ ± 0.1	-	-	-	-	0 ^a^	0 ^a^
Linalool	*P. orientalis* P49	0 ^a^	-	-	-	-	0 ^a^	0 ^a^
	*P. orientalis* P110	8.0 ^c^ ± 1.6	90.9 ^b^ ± 8.8	-	78.5 ^b^ ± 3.7	-	46.8 ^b^ ± 8.9	0 ^a^
Citral	*P. orientalis* P49	0 ^a^	-	-	-	-	0 ^a^	0 ^a^
	*P. orientalis* P110	0 ^a^	-	-	-	-	0 ^a^	0 ^a^

(-)—MIC > 90.9 µL/mL (36 °C, 24 h) or 166.7 µL/mL (15 °C, 72 h). ^a–d^ Distinct letters within the same column indicate significant difference in the same experiment and the same bacteria strain (*p* < 0.05).

**Table 2:** The range of bioactive compounds [%] in tested essential oils determined by gas-chromatography.

Bioactive Compound/Oil Type	Rosemary [%]	Bitter Orange [%]	Black Pepper [%]	Bergamot [%]	Nutmeg [%]	Lemongrass [%]	Lime [%]	Juniper [%]
α-pinene	19.0 ± 1.0	0.6 ± 0.2	10.0 ± 2.0	nd	17.5 ± 1.5	nd	1.75 ± 0.75	1.75 ± 0.75
Camphene	3.0 ± 1.0	0.5 ± 0.3	nd	nd	nd	nd	nd	nd
ß-pinene	9.5 ± 1.5	0.08 ± 0.07	9.5 ± 2.5	7.5 ± 2.0	14.5 ± 1.0	nd	21.5 ± 3.5	21.5 ± 3.5
Myrcene	1.75 ± 0.25	2.25 ± 0.75	nd	nd	2.2 ± 0.4	nd	1.75 ± 0.75	1.75 ± 0.75
Limonene	3.0 ± 0.5	94.5 ± 1.5	13.5 ± 3.5	34.5 ± 4.5	nd	1.85 ± 0.65	nd	nd
Cineol	19.0 ± 1.0	nd	nd	nd	nd	nd	nd	nd
*p*-cymene	1.6 ± 0.6	nd	nd	nd	nd	nd	nd	nd
Camphor	20.5 ± 0.5	nd	nd	nd	nd	nd	nd	nd
Bornyl acetate	0.7 ± 0.2	nd	nd	nd	nd	nd	nd	nd
α-terpineol	1.3 ± 0.3	nd	nd	nd	5.0 ± 1.0	nd	nd	nd
Borneol	3.75 ± 0.75	nd	nd	nd	nd	nd	nd	nd
Verbenone	1.2 ± 0.5	nd	nd	nd	nd	nd	nd	nd
Linalool	nd	0.42 ± 0.28	nd	12.0 ± 3.0	nd	nd	nd	nd
Decanal	nd	0.40 ± 0.30	nd	nd	nd	1.6 ± 0.1	nd	nd
Neral	nd	0.22 ± 0.19	nd	nd	nd	30.0 ± 2.0	nd	nd
Geranial	nd	0.13 ± 0.08	nd	0.36 ± 0.13	nd	37.0 ± 2.0	nd	nd
ß-sinensal	nd	0.21 ± 0.20	2.0 ± 1.5	nd	nd	nd	nd	nd
Delta-3-Carene	nd	nd	10.5 ± 1.5	nd	1.75 ± 0.95	nd	nd	nd
ß-Caryophyllene	nd	nd	24.5 ± 4.5	nd	nd	nd	nd	nd
Linalyl acetate	nd	nd	28.0 ± 6.0	nd	nd	nd	nd	nd
γ-terpinene	nd	nd	nd	8.0 ± 2.0	4.4 ± 0.8	nd	21.5 ± 3.5	21.5 ± 3.5
β-bisabolene	nd	nd	nd	0.43 ± 0.13	nd	nd	nd	nd
Sabinene	nd	nd	nd	nd	18.0 ± 1.0	nd	nd	nd
Citronellol	nd	nd	nd	nd	nd	nd	nd	nd
Eugenol	nd	nd	nd	nd	0.50 ± 0.01	nd	nd	nd
Myristicin	nd	nd	nd	nd	9.5 ± 1.5	nd	nd	nd
α-thujene	nd	nd	nd	nd	1.85 ± 0.25	nd	nd	nd
Methyl heptanone	nd	nd	nd	nd	nd	1.05 ± 0.95	nd	nd
Citral	nd	nd	nd	nd	nd	68.5 ± 1.5	10.0 ± 5.7	10.15 ± 5.85
Geranyl acetate	nd	nd	nd	nd	nd	2.0 ± 1.5	nd	nd

nd—not detected.

**Table 3:** The percentage of bacteria in three metabolic fractions and the distribution of individual cell fractions in the control probe of *P. orientalis* P49 and *P. orientalis* P49 bergamot oil.

Essential Oil	*P. Orientalis* Strain	Low Metabolic Activity [%] 36 °C, 24 h	Low Metabolic Activity [%] 15 °C, 72 h	Medium Metabolic Activity [%] 36 °C, 24 h	Medium Metabolic Activity [%] 15 °C, 72 h	High Metabolic Activity [%] 36 °C, 24 h	High Metabolic Activity [%] 15 °C, 72 h
control probe	P49	7.1 ^a^ ± 0.2	2.3 ^a^ ± 0.3	7.2 ^a^ ± 0.1	9.2 ^a^ ± 0.6	67.8 ^c^ ± 0.7	80.1 ^c^ ± 0.8
	P110	10.0 ^a^ ± 0.4	1.4 ^a^ ± 0.2	15.0 ^b^ ± 0.5	4.2 ^a^ ± 0.1	65.0 ^c^ ± 0.3	86.1 ^b^ ± 0.6
black pepper	P49	10.3 ^a^ ± 0.1	2.2 ^a^ ± 0.2	8.2 ^a^ ± 0.6	6.8 ^a^ ± 1.2	65.9 ^c^ ± 0.4	83.1 ^c^ ± 2.2
	P110	11.8 ^a^ ± 0.4	2.0 ^a^ ± 0.1	7.9 ^a^ ± 0.1	2.5 ^a^ ± 0.2	54.9 ^c^ ± 0.6	91.1 ^c^ ± 0.2
lemongrass	P49	29.0 ^b^ ± 0.3	36.7 ^b^ ± 0.5	13.2 ^b^ ± 0.2	24.6 ^c^ ± 0.6	47.8 ^b^ ± 0.5	25.9 ^a^ ± 0.4
	P110	52.^b^ ± 0.7	6.8 ^a^ ± 0.5	10.6 ^a^ ± 0.1	5.8 ^a^ ± 0.4	6.4 ^a^ ± 0.2	78.3 ^a^ ± 0.5
Juniper	P49	9.2 ^a^ ± 0.2	4.2 ^a^ ± 0.1	8.1 ^a^ ± 0.2	18.1 ^b^ ± 2.6	67.3 ^c^ ± 1.0	62.8 ^b^ ± 3.3
	P110	16.4 ^a^ ± 0.2	2.7 ^a^ ± 0.1	10.1 ^a^ ± 0.1	6.0 ^a^ ± 0.6	46.8 ^b^ ± 0.3	82.4 ^b^ ± 1.1
bergamot	P49	31.2 ^b^ ± 0.4	2.5 ^a^ ± 0.3	9.2 ^a^ ± 0.5	11.7 ^b^ ± 1.0	29.8 ^a^ ± 0.9	73.2 ^b^ ± 1.0
bitter orange		5.2 ^a^ ± 0.1	3.8 ^a^ ± 0.7	5.3 ^a^ ± 0.2	15.8 ^b^ ± 1.4	60.4 ^c^ ± 1.2	72.1 ^a^ ± 1.1
Lime	P110	23.0 ^b^ ± 0.2	5.3 ^a^ ± 0.6	7.6 ^a^ ± 0.4	9.2 ^a^ ± 0.7	25.0 ^a^ ± 0.8	73.3 ^b^ ± 0.4
rosemary		47.8 ^b^ ± 0.6	3.2 ^a^ ± 0.3	10.6 ^a^	9.5 ^a^ ± 0.3	0.2 ^a^ ± 0.1	75.6 ^a^ ± 0.7

^a–c^ Distinct letters within the same column indicate significant difference in the same experiment and the same bacteria strain (*p* < 0.05).
